# Allocation strategy of nonstructural carbohydrates in *Spiraea* L. across different grassland types in the Altai Mountains

**DOI:** 10.3389/fpls.2025.1562363

**Published:** 2025-06-06

**Authors:** Xuexi Ma, Lianlian Fan, Meiniu Yang, Jiangyue Li, Meng Yan, Ziyue Yang, Xi Chen, Bo Zhang, Yaoming Li, Yingzhi Gao

**Affiliations:** ^1^ Key Laboratory of Ecological Safety and Sustainable Development in Arid Lands, Xinjiang Institute of Ecology and Geography, Chinese Academy of Sciences, Urumqi, China; ^2^ College of Grassland Science, Xinjiang Agricultural University, Key Laboratory of Grassland Resources and Ecology of Western Arid Desert Area of the Ministry of Education, Urumqi, China; ^3^ Chinese Academy of Sciences (CAS) Research Center for Ecology and Environment of Central Asia, Urumqi, China; ^4^ Bayinbuluk Alpine Grassland Observation and Research Station of Xinjiang, Bayinbuluk, Xinjiang, China; ^5^ Institute of Grassland Science, Northeast Normal University, Key Laboratory of Vegetation Ecology of the Ministry of Education, State Environmental Protection Key Laboratory of Wetland Ecology and Vegetation Restoration, Changchun, China

**Keywords:** grassland shrub encroachment, Altai Mountains, nonstructural carbohydrates, soluble sugars, starch

## Abstract

Global climate change and overgrazing have exacerbated shrub encroachment in arid and semi-arid grasslands and risking the stability and multifunctionality of grassland ecosystems. This shift between shrub and grassland raises a great concern to explicitly analyze the distribution of shrubs into arid and semi-arid grasslands and related environmental adaptation by using their nonstructural carbohydrates (NSCs) concentrations, and assess their allocation strategies and key determinants of these strategies. This research study was conducted in the Altai mountains of northwest of China and specifically focused on *Spiraea*, a dominant shrub in this region, and analyzed the NSCs component concentrations and their allocation in *Spiraea* leaves across different grassland types. The results showed significant variations in the NSCs concentrations, components, and allocation in *Spiraea* leaves across various grassland types, demonstrating higher overall values in meadows rather than grasslands (*p* < 0.05). Moreover, the total NSCs concentrations(the sum of soluble sugar and starch concentrations, and the ratio of soluble sugar to starch) exhibited consistent responses to environmental changes, and indicated increasing trends with elevation, soil water content (SWC), and soil total nitrogen concentrations (TN), while decreasing with mean annual temperature (MAT). Notably, MAT and SWC were emerged as the factors with the highest influence on NSCs concentrations and allocation in *Spiraea* leaves. These findings indicate that *Spiraea* in the Altai mountains can rapidly adapt to environmental changes across different grassland types by calibrating their NSCs concentrations and composition. These findings also present an insightful theoretical foundation for managing shrub-grassland encroachments in the Altai mountains region and other arid and semi-arid regions.

## 1 Introduction

Nonstructural carbohydrates (NSCs) are crucial energy sources and osmotic adjustment substances in plants that sustain plant growth and facilitate survival under stressful conditions (Hartmann and Trumbore, 2016; Yin et al., 2021)., NSCs include starch (energy storage) and soluble sugars (direct energy and osmotic adjustment) These two components can interconvert under specific environmental conditions to help plants adapt to diverse environments (McDowell, 2011; Zhang et al., 2023). For instance, under similar growth conditions, plants with higher NSCs concentrations tend to have higher chances of survival and better ability to do so (Blumstein et al., 2023; O’Brien et al., 2014). Therefore, NSCs storage can shield plants against carbon supply deficiencies, allowing them to cope with abiotic and biotic stresses. Consequently, NSCs storage is an important physiological indicator for assessing plant responses to environmental changes (Barker Plotkin et al., 2021; Martínez-Vilalta et al., 2016). Thus, explaining not only the concentrations of starch and soluble sugars within plants but also their allocation strategies is necessary to understand how plants adapt to diverse environmental conditions.

The factors influencing NSCs concentrations and allocation strategy include precipitation, temperature, and soil physicochemical properties (Guo et al., 2020; O’Brien et al., 2014; Zhang et al., 2024a). Increased drought intensity and duration correspond with a decline in the starch content in most woody plant organs and a rise in the concentration of soluble sugars, causing an increase in the ratio of soluble sugar to starch. Consequently, plants convert more starch into soluble sugars for osmotic adjustment, thereby maintaining cell osmotic pressure and ensuring plant growth and survival under drought conditions (He et al., 2020; Liu et al., 2019). Similarly, plants demonstrate analogous responsive characteristics in response to temperature stress, where high or low temperatures trigger starch hydrolase activity, prompting the conversion of starch into soluble sugars (Thalmann and Santelia, 2017; Yin and Zhang, 2016). In their study on alpine coniferous forests in the Qinghai–Tibet Plateau, Zhang et al. (2024a) argued that plant NSCs, especially those found in aboveground leaves, are mainly influenced by the combined effects of temperature and precipitation. Along with hydrothermal conditions, soil nutrient supply is a key factor affecting NSCs concentrations and allocation in plants. As a result of continuous climate changes and shifts in regional precipitation patterns, northwestern China is transitioning toward a warmer and wetter climate. In this context, soil nitrogen and phosphorus supply can promote plant growth and stressor resistance, thereby compensating for any adverse conditions such as drought and, subsequently, influencing NSCs concentrations and dynamics in plants (Zhang et al., 2024b; Zhou et al., 2011). Therefore, the concentration and allocation dynamics of plant NSCs demonstrate strong species- and region-specific characteristics.

The Altai mountains are a transitional zone between the Taiga biogeographical community and the arid desert biogeographical community. This adds to the significance of their grasslands as far as northern China is concerned. However, human activities such as overgrazing have contributed to severe degradation of the grassland ecosystem in the Altai mountains (Li et al., 2024). Moreover, the coverage and diversity of herbaceous plants in the grassland have steadily decreased. In contrast, the coverage of shrubs has rapidly increased, gradually replacing herbaceous plants as the dominant species in the region, causing grassland shrub encroachment (Ding and Eldridge, 2023; Moore et al., 2020; Ma and Gao, 2025). This has reduced the grassland ecosystem’s productivity, carbon sequestration capacity, nutrient cycling, and water-holding capacity, threatening its multifunctionality (Eldridge et al., 2011; Pierce et al., 2019). The shrub species of the genus *Spiraea*, with their developed root systems and better drought and nutrient-poor tolerance than those of herbaceous plants, are widely distributed in the Altai mountains grassland. They have become the dominant species in the region’s grassland shrub encroachment. However, the exact mechanisms underlying the adaptation of *Spiraea* to the Altai mountains’ diverse climatic conditions in terms of NSCs composition and allocation remain shrouded in uncertainty shrouded in concentration and their response to environmental gradients are poorly understood. Hence, the present study proposed two scientific hypotheses. First, a decrease in precipitation and temperature will cause a corresponding decrease in plant starch concentration and a corresponding increase in the ratio of soluble sugar to starch. Second, how do climatic and edaphic variables across grassland types influence the NSC allocation strategy of Spiraea, and which factors most strongly drive variation? To validate these hypotheses, this study selected the Altai mountains as the research area and focused on the dominant shrub species, *Spiraea*, as the subject of investigation. By systematically collecting samples of *Spiraea* from various grassland types and thoroughly analyzing the NSCs concentrations and proportions in their leaves, the study aimed to explore the NSCs concentrations and allocation patterns within *Spiraea* foliage under different environmental conditions. Further, it attempted to identify the key factors influencing NSCs in *Spiraea* leaves, thus presenting crucial scientific evidence for the stability and informed management of grassland ecosystems amid global climate change. This study is the first to quantify leaf NSC concentrations in Spiraea across Altai mountain grasslands, revealing how shrub encroachment influences ecosystem responses to climate change.

## 2 Materials and methods

### 2.1 Study area

The Altai mountains, which stretch nearly 1,200 km in a northwest direction along the borders of Russia, China, and Mongolia, reach a maximum elevation of 4,374 m. The Chinese segment of the Altai mountains (44°11’~46°20’N, 84°31’~90°00’E) is situated in the heart of the Eurasian continent. It occupies the middle southern slope of the mountain range, running in a northwest–southeast direction and gradually descending. This region is a significant climatic and natural boundary influenced by the Pacific and Atlantic Oceans that is marked by a continental temperate climate, boasting cold winters and abundant snowfall. The annual precipitation averages around 300 mm, demonstrating a vertical zonation pattern ranging from 100 mm in the lower mountainous regions to 600 mm in the higher alpine areas. Additionally, the annual mean temperature varies between -3.6°C and 1.8°C (Huang et al., 2015).

The Altai mountains region demonstrates complete vertical vegetation zonation, with the grassland zone primarily condensed at elevations between 800 and 1,800 m. Recently, the grasslands in this region have revealed a marked tendency toward shrub encroachment in response to global climate change and overgrazing. In this context, the dominant shrubs belong to the genus *Spiraea* (*Spiraea salicifolia* L.), including species such as *Spiraea hypericifolia* L., *Spiraea media* Schmidt, and *Lonicera japonica* Thunb (Ma et al., 2025).

### 2.2 Experimental design

**Figure 1:**
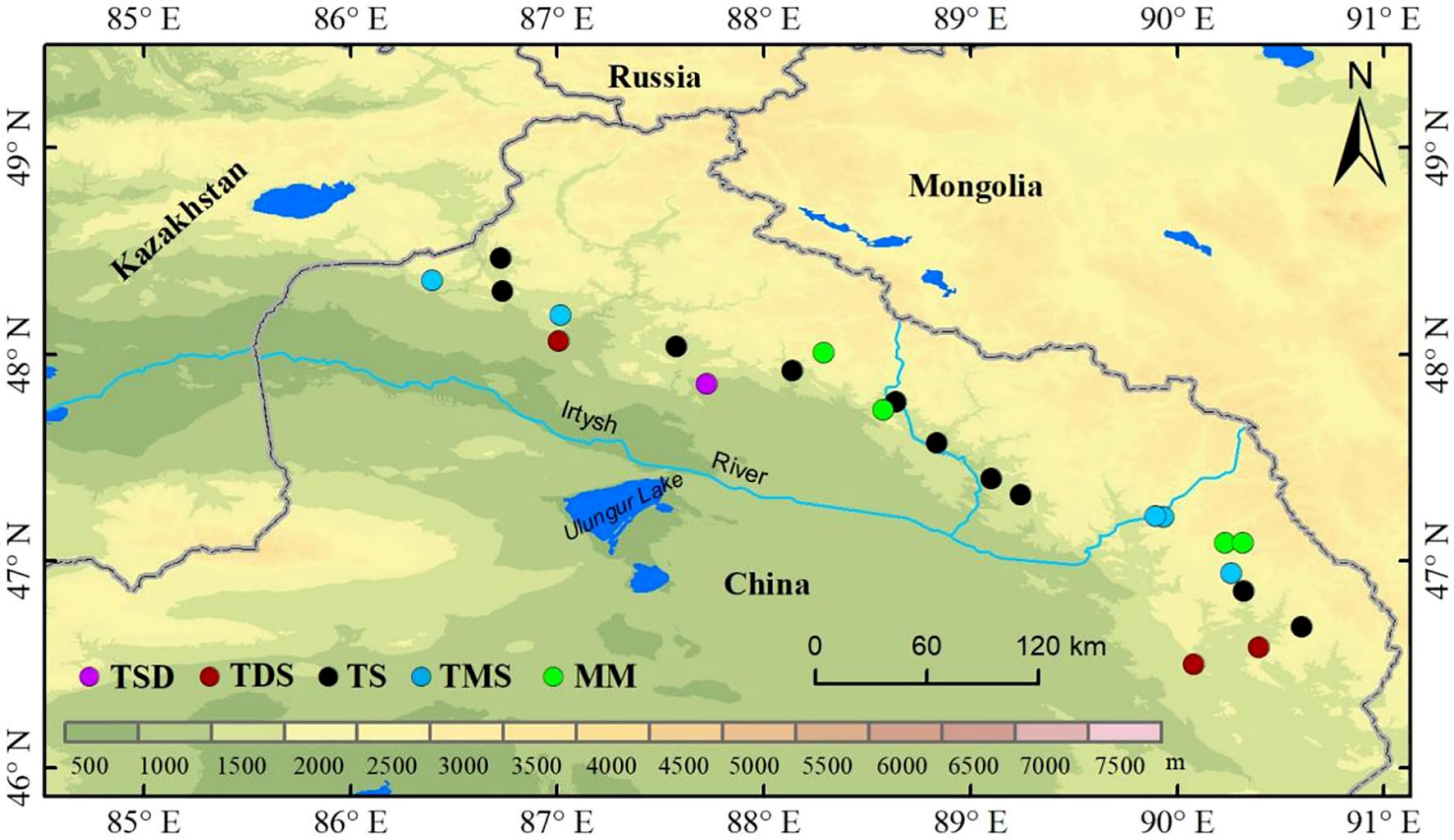
Distribution map of sample points.

In July 2023, we collected leaves from five healthy *Spiraea* plants across different grassland types in the Altai mountains (**Figure 1**). These grassland types included temperate steppe desert (TSD), constituting one site; temperate desert steppe (TDS), constituting three sites; temperate steppe (TS), constituting 10 sites; temperate meadow steppe (TMS), constituting five sites; and mountain meadow (MM), constituting four sites (**Figure 1**). The mean annual precipitation (MAP) gradient across the sampling sites ranged from 155 mm to 354 mm, whereas the mean annual temperature (MAT) gradient ranged from -2.92°C to 5.31°C. For each sampling site, a large plot of 100 m × 100 m was established, within which five small plots of 10 m × 10 m were set up following a five-point sampling method. Further, within each small plot, leaves from five healthy *Spiraea* plants were randomly collected between 11:00 and 16:00 to minimize diurnal variation in NSC concentrations and mixed to form one sample, with five replicate samples in total. We transported samples to the laboratory and fixed them at 105°C for 10 minutes. Subsequently, they were dried at 65°C to constant weight before being pulverized in a ball mill for future use.

Concurrently, soil samples were collected from the 0–10 cm layer using a soil auger within each small plot at each site, with five replicates per site. The collected soil samples were sieved through a 2 mm mesh to remove large debris or roots. Then, they were divided into two portions. While one was air-dried to determine soil organic carbon (SOC), total nitrogen (TN), total phosphorus (TP), available phosphorus (AP), pH, and electrical conductivity (EC), the other was refrigerated at 4°C and analyzed for soil water content (SWC) and available nitrogen (AN) within one week.

### 2.3 Determination of NSCs concentrations

Primarily, NSCs comprise starch and total soluble sugars, as these two components account for over 90% of the total NSCs concentrations. Therefore, in the present study, the total NSCs concentrations were calculated as the sum of soluble sugars and starch—both of which were measured through the traditional anthrone–sulfuric acid method (Zhang et al., 2023). Using this method, 0.15 g of dried and powdered plant leaf samples were accurately weighed and placed in 10 mL of 80% absolute ethanol. Afterward, the mixture was extracted in a boiling water bath for 10 minutes. This mixture was then centrifuged at 4,000 r·min^-1^ for 10 minutes to collect the supernatant as the extract of soluble sugars. Subsequently, 10 mL of 30% (v/v) perchloric acid was added to the centrifuged precipitate, and the mixture was allowed to stand overnight. After accurate extraction in a water bath at 80°C for 10 minutes to ensure complete hydrolysis of starch, the mixture was cooled and centrifuged again at 4,000 r·^-1^ for 10 minutes. Following this, the supernatant was used to determine the starch concentration.

### 2.4 Collection and measurement of environmental factors

The gravimetric method was used to determine SWC. Under this method, fresh soil samples were dried in an oven at 105°C for 48 hours until a constant weight was achieved, followed by weighing. The pH value and EC were measured using the potentiometric method, with soil-to-water ratios of 1:2.5 and 1:5, respectively. Moreover, SOC was determined using the HCl titration-combustion method on a carbon-nitrogen analyzer (Multi 3100C/N, Analytik Jena AG, Germany). Furthermore, using a fully automated flow analyzer, the TN and TP concentrations in the soil were measured after digestion with concentrated sulfuric acid, perchloric acid, and hydrofluoric acid, and AP concentration was extracted with 0.5 mol L^-1^ NaHCO_3_ solution (Bran Luebbe, AA3, Germany). The Kjeldahl method was employed for TN determination, while the molybdenum-antimony anti-colorimetry method was used for both TP and AP. Additionally, the AN concentration in the soil was determined using the alkaline hydrolysis diffusion method (Yin et al., 2024).

### 2.5 Statistical analysis

To begin with, the obtained data were subjected to normality and homogeneity of variance tests. The differences in soluble sugars, starch, soluble sugar/starch ratio, and total NSCs among different grassland types were compared using a one-way analysis of variance (ANOVA), followed by the least significant difference (LSD) test. Additionally, the graphics were created using the ggplot2 package in R 4.2.1; Spearman correlation analysis and random forest modeling were conducted using the randomForest and linkET packages in R 4.1.3 to assess the impact of abiotic factors on soluble sugars, starch, soluble sugar/starch ratio, and total NSCs. Furthermore, regression analyses were performed between key abiotic factors and these NSCs components to identify potential abiotic factors influencing their concentrations. To explain the complex relationships between environmental factors and NSCs in *Spiraea*, a piecewise structural equation model (SEM) was constructed using the “piecewiseSEM” package in R 4.2.1, which was used to determine the direct and indirect effects of climatic factors and soil physicochemical properties on NSCs. Model evaluation was based on Fisher’s C significance (0.05 < *p* < 1) and the AIC, with stepwise refinement of the model as per the significance of path coefficients (Tian et al., 2022). Throughout the analysis and visualization process, various packages were utilized, including “dplyr,” “ggplot2,” “patchwork,” “tidyverse,” and “vegan.”

## 3 Results

### 3.1 Effects of different grassland types on physical and chemical properties of soil

**Figure 2:**
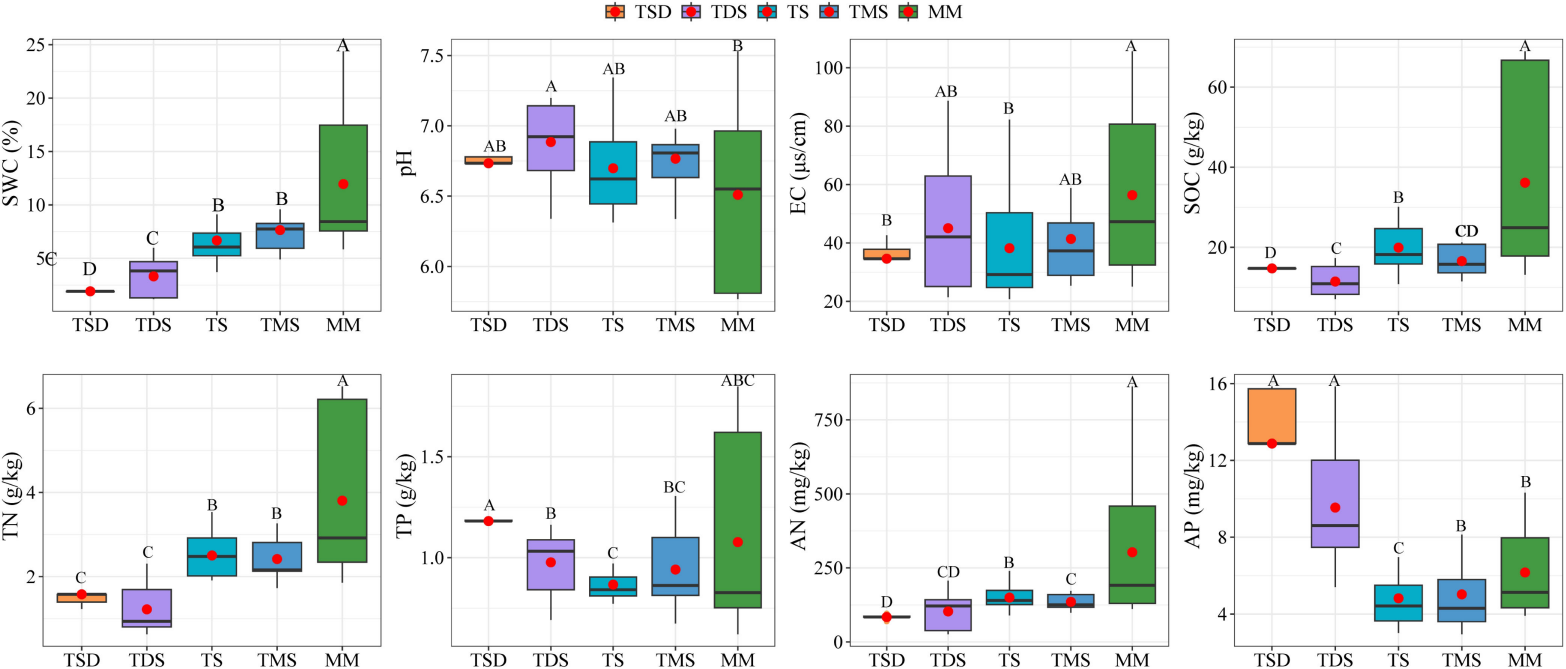
Effects of different grassland types on soil physicochemical properties; the different letters indicate significant differences between treatments at *p* < 0.05.

Notable differences were observed in the soil physicochemical properties across grassland types. The SWC, SOC, TN, and AN levels were higher in TMS and MM soils than in TDS and TS soils (**Figure 2**, *p* < 0.05). Conversely, the soil pH and AP concentrations were significantly lower in TMS and MM soils than in TDS and TS soils (*p* < 0.05).

### 3.2 Characteristics of differences in NSCs concentrations of *Spiraea* across different grassland types

**Figure 3:**
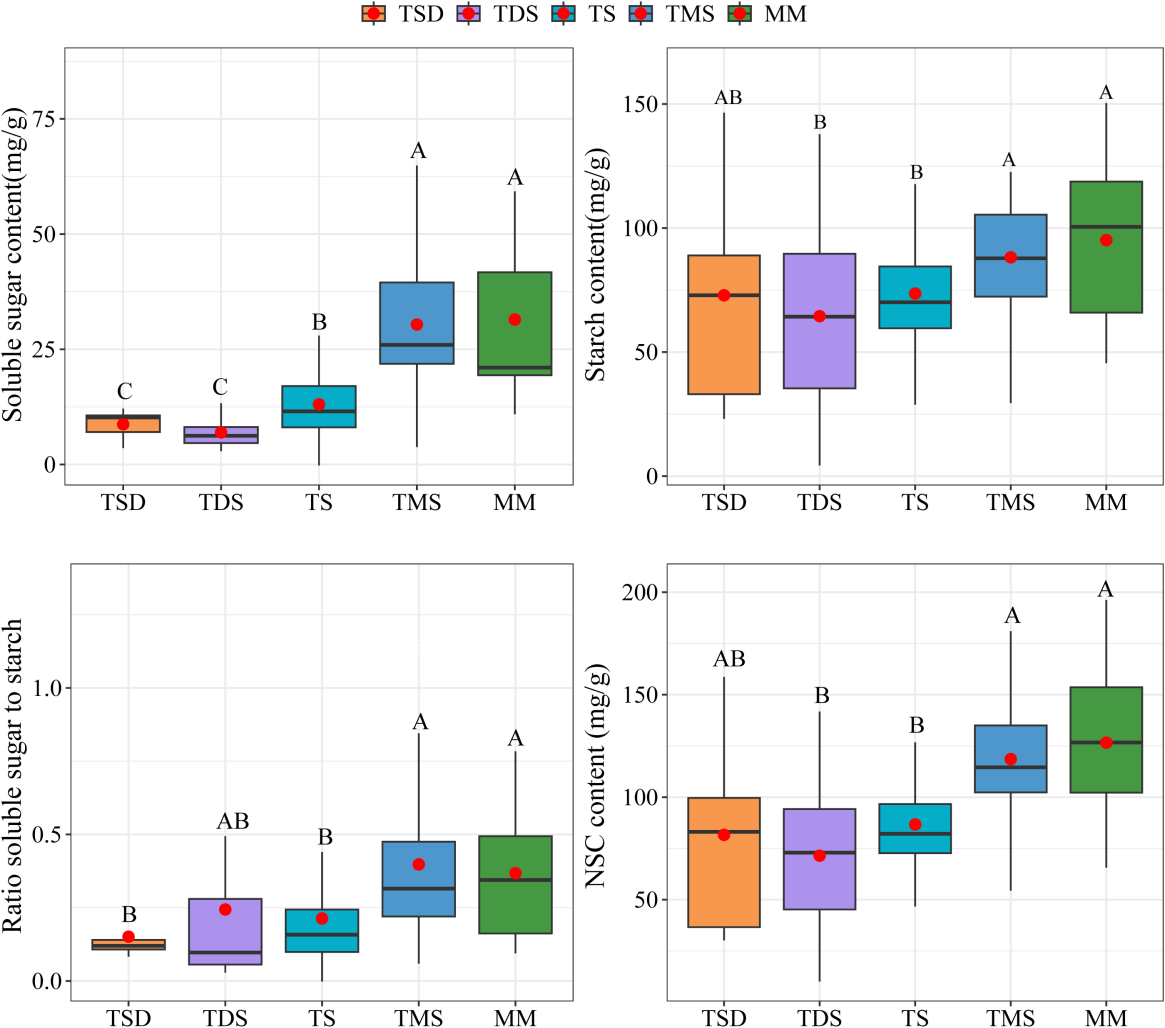
Differences in NSCs and their components of *Spiraea* across various grassland types; different letters indicate significant differences between treatments at *p* < 0.05.

Significant differences were observed in the total soluble sugars, starch, total NSCs concentrations, and the ratio of soluble sugar to starch of *Spiraea* across different grassland types. The soluble sugar, starch, NSCs concentrations, and soluble sugar/starch ratio in *Spiraea* from TMS and MM soils were significantly higher than those from TDS and TS soils (**Figure 3**, *p* < 0.05). Further, the soluble sugar concentration in *Spiraea* from TSD soils was significantly lower than that from TMS and MM soils (*p* < 0.05), while no significant differences were found in starch and NSCs concentrations (*p* > 0.05).

### 3.3 Relationship between NSCs concentrations in *Spiraea* and environmental factors

**Figure 4:**
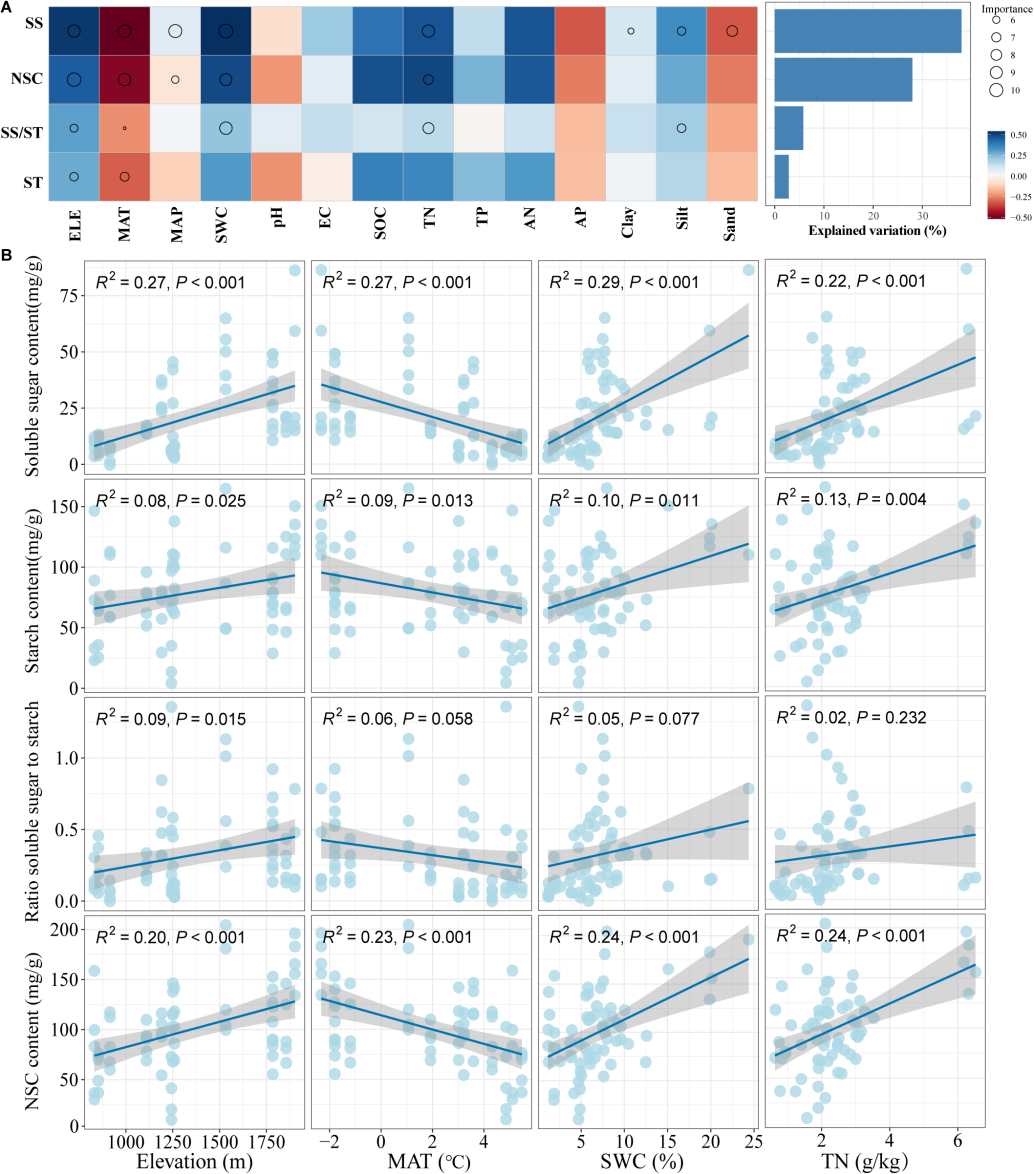
**(A)** Random forest analysis of key factors influencing the concentrations of NSCs and their components in *Spiraea* leaves; **(B)** Relationship between the concentrations of NSCs and their components in *Spiraea* leaves and environmental variables; here, SS represents total soluble sugars, ST represents the starch concentration in *Spiraea* leaves, and ELE represents elevation.

The random forest analysis found that climate and soil environmental factors explained a significantly higher proportion of the variation in total soluble sugar concentration (37.95%) and total NSCs concentrations (27.99%) than starch concentration (2.84%) and the ratio of soluble sugar to starch (5.82%). As seen in **Figure 4A**, elevation, MAT, SWC, and TN were identified as the most important predictors influencing the variation in NSCs and their component concentrations in *Spiraea* leaves (**Figure 4A**). Moreover, as **Figure 4B** illustrates, NSCs and their individual component concentrations in *Spiraea* leaves significantly increased with increasing elevation, SWC, and TN while significantly decreasing with increasing MAT.

### 3.4 Key determinants influencing the NSCs concentrations in *Spiraea*

**Figure 5:**
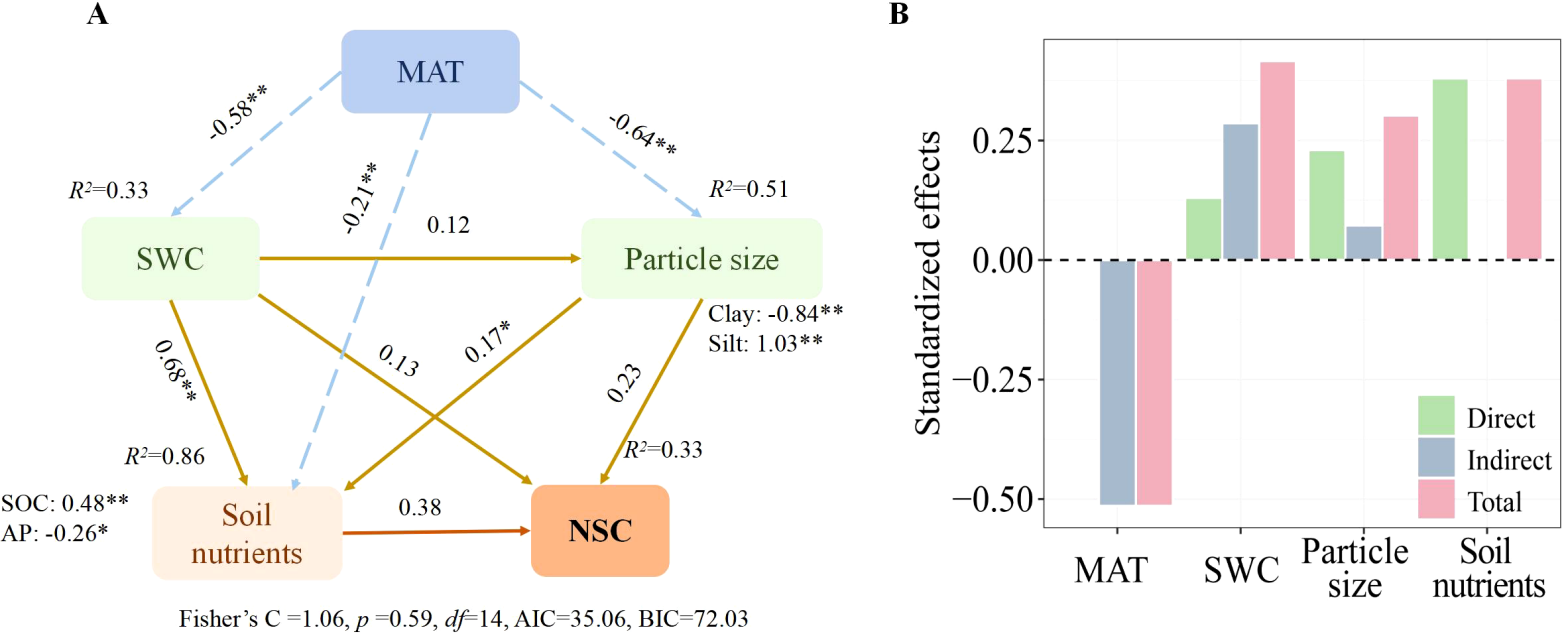
**(A)** A piecewise SEM calculating the direct and indirect effects of climatic factors, soil nutrients, SWC, and soil particle size on the NSCs in *Spiraea*; **(B)** The overall effects of all predictors on NSCs concentrations in *Spiraea*; here, the dashed lines indicate negative effects, while solid lines indicate positive effects; ** and * denote significance levels of *p* < 0.01 and *p* < 0.05, respectively.

As **Figure 5** illustrates, we employed piecewise SEM to analyze how climate, soil particle size, and physicochemical properties directly and indirectly influence the spatial variation of total NSCs in *Spiraea*. The results suggest that MAT, soil particle size, and physicochemical properties collectively explain 33% of the spatial variation in NSCs in *Spiraea*. Notably, MAT emerged as the primary factor influencing the spatial variation of NSCs, exerting a negative effect primarily through its indirect impacts on SWC, soil particle size, and physicochemical properties. Further, SWC had a secondary but significant impact on its spatial variation. In contrast, soil particle size exhibited the least influence.

## 4 Discussion

### 4.1 Influence of hydrothermal conditions on the NSCs concentrations of *Spiraea*

Hydrothermal conditions limit plant productivity and growth in various ecosystem types within arid regions, exerting direct and indirect influences on plant physiological metabolism (Dickman et al., 2019; Zhao et al., 2022). As organs highly sensitive to environmental changes, plant leaves can reflect adaptive responses through timely changes in NSC concentrations. This study revealed that MAT and SWC significantly impact the total NSCs, their individual component concentrations, and the ratio of soluble sugar to starch in different grassland types of the Altai Mountains, which aligns with our hypothesis. Under the higher SWC conditions, *Spiraea* can enhance its metabolic activities and carbon reserve synthesis by acquiring more water, thereby increasing the concentrations of soluble sugars and starch (O’Brien et al., 2014; Zhang et al., 2024a). Moreover, a meta-analysis indicates that NSCs accumulate under moderate water stress, whereas severe or prolonged drought may cause net carbon reserve losses (He et al., 2020). This suggests that along with altitudinal gradients, plants balance temperature and water availability. This trade-off in resource utilization determines the distribution and concentration variations of carbohydrates in *Spiraea*.

As the factor limiting plant growth in arid regions the most, water directly influences photosynthesis, as well as the synthesis and allocation of NSCs in plants (Adams et al., 2017; Sevanto et al., 2014). Precipitation in mountainous areas is more unevenly distributed than in plain ecosystems, leaving plants more susceptible to water limitations during their growth process. Subsequently, this affects photosynthetic carbon fixation. Generally, plants growing in arid environments retain high NSCs reserves and allocate a larger proportion of these reserves as soluble sugars, thus reducing water potential during turgor loss and promoting their survival during droughts (Wiley and Helliker, 2012). Our results indicate that the NSCs concentrations in *Spiraea* are influenced more by SWC than by precipitation, which contradicts both our hypothesis and previous findings. This discrepancy is caused by the region’s soil texture and topographical environment, where precipitation in mountainous areas is more vulnerable to runoff and less likely to infiltrate the soil. Notably, SWC is a crucial factor here as it is more accessible to plants. An increase in SWC corresponds with an increase in the total NSCs concentrations and its components in *Spiraea* plants. Under adequate water conditions, Spiraea efficiently fixes soluble sugars and starch through photosynthesis, likely as a strategy to endure harsh winter periods (Jang and Sheen, 1994; Yin et al., 2021).

Temperature exerts a more significant influence than SWC on the composition of NSCs in different grassland types of the Altai Mountains. This may be because of the regional climate, where the Altai Mountains, located at a higher latitude and in a mountainous area, receive more precipitation (approximately 300 mm) than other arid grasslands at similar latitudes. Further, the lower temperatures lead to slower water evaporation and higher water availability. Several studies have found that plant carbon allocation efficiency and growth rate are temperature-dependent, especially in high-latitude regions (García-Carreras et al., 2018). Moreover, soluble sugars, as important energy sources and osmotic adjustment substances in plants, amass in response to environmental stresses such as low temperatures, thereby enhancing the plant’s resistance (Rodríguez-Calcerrada et al., 2011; Klopotek and Kläring, 2014). Therefore, as temperatures decrease, *Spiraea*, under low-temperature stress, increases its NSCs concentrations, particularly soluble sugars, to elevate leaf osmotic pressure. Additionally, they secrete starch-hydrolyzing enzymes to hydrolyze part of the starch into soluble sugars, thus maintaining normal metabolic activities (Yin and Zhang, 2016; Michaletz, 2018; He et al., 2023).

### 4.2 Impact of grassland type on the NSCs concentrations of *Spiraea*

This study’s findings validate our hypothesis that different grassland types significantly impact the NSCs and their component concentrations in *Spiraea*. We found that the concentrations of soluble sugars and total NSCs in *Spiraea* from high-altitude MMs and TMSs are significantly higher than those in other grassland types. This variation may be because of the low-temperature conditions at higher altitudes, which slow down plant metabolic rates (Körner, 2007), reducing the rate of carbon reserve decomposition. Simultaneously, the higher SWC and nutrient supply (such as SOC and TN) enhance plant photosynthetic efficiency and promote carbohydrate accumulation (Hoch and Körner, 2009; Blumstein et al., 2023). Conversely, in low-altitude TDSs and TSDs, plants experience greater environmental stress due to higher temperatures and the scarcity of SWC and nutrients. Moreover, the more frequent utilization of their carbohydrates for maintaining basic metabolism and survival adaptations results in significantly lower NSCs concentrations, with the lowest soluble sugar/starch ratio (Moore et al., 2021; Ryhti et al., 2022). This suggests that changes in altitude, hydrothermal conditions, and soil nutrients jointly drive significant differences in the resource allocation strategies of *Spiraea*, gradually transitioning from a rapid growth mode to an adaptation mode focused on storage.

Differences in grassland types significantly alter soil physicochemical properties, indirectly influencing the variation in plant NSCs and their component concentrations (Zhang et al., 2023), with SOC and TN being especially important in this process. While SOC functions as a carbon source for soil microbial activity and plant metabolism that enhances the plant’s carbon fixation and storage capacity (Huang et al., 2024), nitrogen is a key limiting factor for plant growth in arid and semi-arid regions that promotes NSCs accumulation by enhancing plant nitrogen uptake and protein synthesis capabilities, which, in turn, increases enzyme activity related to photosynthesis and carbon metabolism. This promotes water utilization and transport, enhancing carbon utilization strategies (Schimel et al., 2004; Garnett et al., 2009; Wang et al., 2022). This was further confirmed in the present study, where SEM revealed that soil nitrogen significantly influences the NSCs concentrations in *Spiraea* that grows in the Altai Mountains. As soil TN content increases, there is a corresponding increase in plant soluble sugars, total NSCs, and the ratio of soluble sugar to starch. This trend reflects nitrogen supply, which alleviates limitation in plants, enhancing photosynthetic activity and promoting the accumulation of soluble sugars and other substances (Zhou et al., 2011; Xie et al., 2018). Furthermore, the availability of these nutrients is not only related to their concentration but also regulated by soil particle composition, such as the proportion of clay and sand particles. A high proportion of clay particles aids in nutrient retention, whereas a high concentration of sand particles may lead to nutrient loss, particularly in mountainous regions (Wang et al., 2020). Therefore, soil nutrients directly influence plant physiological metabolic processes and resource allocation strategies, driving the dynamic changes in NSCs and their components in *Spiraea*.

## 5 Conclusion

*Spiraea* in the Altai Mountains can adapt to diverse grassland environments by regulating the concentrations and composition of NSCs. This study found that SWC, MAT, soil TNC, and altitude can directly or indirectly affect NSCs concentrations and distribution. Among these factors, MAT and SWC most significantly influence the NSCs concentrations and components of *Spiraea* in different grassland types. As MAT decreases and SWC and soil TNC increase, the soluble sugars, starch, and total NSCs concentrations in *Spiraea* leaves show an upward trend. Thus, these findings offer physiological and ecological insights into why *Spiraea* has become a dominant species in the grassland shrub encroachment of this region, offering theoretical support for scientifically managing grassland shrub encroachment in this area. However, further research is necessary to investigate the NSCs allocation strategies of *Spiraea* above and below ground, as well as their variation patterns during different growth stages.

## Data Availability

The original contributions presented in the study are included in the article/supplementary material. Further inquiries can be directed to the corresponding authors.
